# Angiotensin II-Induced Mesangial Cell Damage Is Preceded by Cell Membrane Permeabilization Due to Upregulation of Non-Selective Channels

**DOI:** 10.3390/ijms19040957

**Published:** 2018-03-23

**Authors:** Gonzalo I. Gómez, Paola Fernández, Victoria Velarde, Juan C. Sáez

**Affiliations:** 1 Departamento de Fisiología, Facultad de Ciencias Biológicas, Pontificia Universidad Católica de Chile, Alameda #340, 8331150 Santiago, Chile; paolafernandezbq@gmail.com (P.F.); vvelarde@bio.puc.cl (V.V.); 2 Instituto Milenio, Centro Interdisciplinario de Neurociencias de Valparaíso, Universidad de Valparaíso, 2381850 Valparaíso, Chile

**Keywords:** connexin hemichannel, gap junction, P2X_7_ receptor, pannexin1 channel, extracellular ATP, oxidative stress, angiotensin receptors, Fasudil, Y-27632

## Abstract

Connexin43 (Cx43), pannexin1 (Panx1) and P2X_7_ receptor (P2X_7_R) are expressed in kidneys and are known to constitute a feedforward mechanism leading to inflammation in other tissues. However, the possible functional relationship between these membrane channels and their role in damaged renal cells remain unknown. In the present work, we found that MES-13 cells, from a cell line derived from mesangial cells, stimulated with angiotensin II (AngII) developed oxidative stress (OS, thiobarbituric acid reactive species (TBARS) and generated pro-inflammatory cytokines (ELISA; IL-1β and TNF-α). The membrane permeability increased progressively several hours before the latter outcome, which was a response prevented by Losartan, indicating the involvement of AT1 receptors. Western blot analysis showed that the amount of phosphorylated MYPT (a substrate of RhoA/ROCK) and Cx43 increased progressively and in parallel in cells treated with AngII, a response followed by an increase in the amount in Panx1 and P2X_7_R. Greater membrane permeability was partially explained by opening of Cx43 hemichannels (Cx43 HCs) and Panx1 channels (Panx1 Chs), as well as P2X_7_Rs activation by extracellular ATP, which was presumably released via Cx HCs and Panx1 Chs. Additionally, inhibition of RhoA/ROCK blocked the progressive increase in membrane permeability, and the remaining response was explained by the other non-selective channels. The rise of activity in the RhoA/ROCK-dependent pathway, as well as in Cx HCs, P2X_7_R, and to a minor extent in Panx1 Chs led to higher amounts of TBARS and pro-inflammatory cytokines. We propose that AngII-induced mesangial cell damage could be effectively inhibited by concomitantly inhibiting the RhoA/ROCK-dependent pathway and one or more non-selective channel(s) activated through this pathway.

## 1. Introduction

In chronic kidney disease, hypertension is one of the most common complications that generate predispositions to other health problems, affecting several organs. Hypertensive nephropathy begins in the glomerulus by increasing intraglomerular pressure. These early events activate and damage mesangial cells, epithelial cells and podocytes in the glomerulus. In turn, these cells produce vasoactive and pro-inflammatory agents, which increase cell damage and promote fibrosis, reducing renal blood flow, permeability and, eventually, glomerular filtration [1].

The renin-angiotensin-system is the prototype of a classic systemic endocrine network, whose actions in the kidney and the adrenal gland regulate blood pressure, intravascular volume and electrolyte balance [2]. Angiotensin II (AngII), a vasoactive peptide found in the renal tissue, stimulates aldosterone secretion, cellular infiltration, proliferation and migration, thrombosis, reactive oxygen species (ROS) production, and contributes to the induction of inflammatory responses common in nephropathy [1].

AngII interacts with AT1 and AT2 receptors, which are coupled to G-protein-dependent pathways. The AT1 receptor activates small G proteins, including Ras, Rac1, RhoA and Rho kinase (ROCK) [3], whereas the AT2 receptor conversely inhibits RhoA [4]. The family of small Rho GTPases (Rho GTPases) plays an important role in AngII signaling that promotes numerous cell changes, including cytoskeletal and gene expression changes [3]. Among the Rho GTPases are Rac1, Cdc42 and RhoA. ROCK, which is an effector downstream of RhoA, is a serine-threonine kinase of ~160 kDa that exists in mammals in two isoforms: ROCK1 and ROCK2 [5,6].

The RhoA/ROCK pathway has received considerable attention due to its involvement in a wide variety of pathological states [6]. Recent studies have revealed that the RhoA/ROCK pathway is involved in regulating the generation of pro-inflammatory cytokines such as TNF-α and IL-1β [3,7]. In addition, it has been proposed that the RhoA/ROCK pathway may play an important role in renal fibrosis by enhancing signaling pathways, which are activated by transforming growth factor-β (TGF-β) and AngII, hence resulting in the activation of nuclear factor-κB (NFκB). Since the discovery of Fasudil and Y-27632, two selective inhibitors of RhoA and ROCK have generated great interest as important regulators in diverse models of kidney damage [6,8]. These inhibitors prevent kidney damage by reducing the expression of extracellular matrix genes, oxidative stress (OS), pro-inflammatory cytokines and macrophage infiltration [3]. Thus, both RhoA and ROCK inhibitions have been proposed as treatments to prevent hypertension and kidney damage [7,9].

Inflammation is a complex process, and it has recently been related to disorders in intercellular communication mediated by connexin gap junctions and hemichannels (Cx GJs and Cx HCs, respectively) [10] as well as pannexin channels (Panx Chs) [11]. GJs are clusters of intercellular channels, each resulting from docking two HCs or connexons at cell-cell interfaces. A HC is constituted by the oligomerization of six Cxs. Undocked HCs can be located in the cell surface and can open under physiological and pathological conditions, thus participating in a number of cellular processes [12]. Both GJs and HCs are permeable to ions and small molecules, and while GJs enable communication between contacting cells, HCs allow for exchanges between intra and extracellular milieus.

Around two dozens of human and rodent Cx isoforms have been identified to date [13]. Each Cx is named according to their predicted molecular weight, preceded by the suffix Cx [14]. In cortical astrocytes, IL-1β and TNF-α reduce intercellular communication mediated by GJs and increase the permeability of the cell membranes through connexin43 (Cx43) HCs [15]. The same occurs with GJs and HCs in astrocytes subjected to hypoxia/reoxygenation in the presence of high glucose concentrations [16]. The Panx family on the other hand consists of 3 members (Panx1–3), and all three have been reported to form functional membrane channels (Panx Chs) [17]. A growing body of evidence suggests that Panx Chs are functional under physiological and pathophysiological conditions, enabling the passage of small molecules between the intra- and extracellular environment such as glutamate and ATP. It is assumed that they act exclusively as single membrane pore channels because they are glycosylated at extracellular arginine residues, which would prevent Panx Chs on adjacent cells from forming GJ channels [18].

Extracellular ATP activates P2X7 receptors (P2X_7_Rs), which are permeable to cations, including Na^+^, K^+^ and Ca^2+^ [19]. In turn, activation of P2X_7_R leads to Panx1 Ch opening [20].

The kidneys contribute significantly to the homeostasis of the whole organism, and to fulfill these functions they require the coordinated action of its different cell types, including vascular and tubular cells. This process is poorly understood, but the involvement of intercellular communication is conceivable through Cx-based channels, Panx Chs and P2X_7_Rs. Nine Cxs (Cx26, Cx30.3, Cx31, Cx32, Cx37, Cx40, Cx45 and Cx46) and Panx1 have been found in the kidney [21,22,23], and changes in their amounts have beendetected in several models of renal damage [10,24,25]. P2X_7_Rs have also been detected in the kidney [26]. However, it remains unknown whether Cx- and Panx-based channels as well as P2X_7_Rs interact, and whether they are regulated by AngII. To our knowledge only a few studies have described the participation of Cx HCs in renal damage [27], and no signaling pathway has been clearly associated with these changes. In the present study, we found that AngII increases membrane permeability in MES13-cells via AT1 receptors, as well as the activation of a RhoA/ROCK-dependent intracellular signaling pathway, followed by the up-regulation of three non-selective channels, and the generation of OS and pro-inflammatory cytokines.

## 2. Results

### 2.1. AngII Increases Cell Membrane Permeability of MES-13 Cells

Since Etd^+^ is a membrane-impermeant cationic dye, which becomes fluorescent when intercalated in nucleic acid strands normally found in the intracellular space [28], we used it to evaluate changes in membrane permeability in MES-13 cells under control conditions and after treatment with AngII.

**Figure 1:**
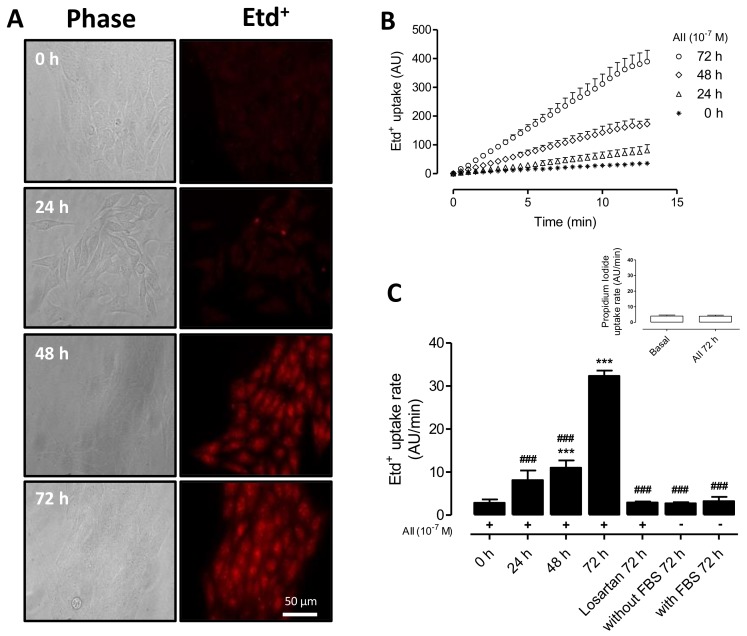
AngII increases the Etd^+^ uptake in MES-13 cells. (**A**) Phase contrast and fluorescence images show MES-13 cells exposed to ethidium (5 μM Etd^+^) for 13 min under AngII stimulation (10^−7^ M) at different periods of time (0, 24, 48 and 72 h). Scale bar = 50 μm; (**B**) Representative time lapse of Etd^+^ uptake in mesangial cells recorded every 30 s, each plotted point corresponds to the mean of 20 cells, mean value ± SE. Dye uptake curve after application of AngII at different times (0, 24, 48 and 72 h); (**C**) Etd^+^ uptake rate of mesangial cells under basal conditions (0 h) or after treatment with AngII for different times (24, 48, and 72 h), with or without FBS (Fetal bovine serum) and Losartan (20 μM). Inset shows propidium iodide uptake rate of mesangial cells under basal conditions or after treatment with AngII for 72 h. Each bar represents the mean value ± SE of ≥3 independent experiments. Statistical significance *** *p* < 0.001 vs. 0 h; ^###^ *p* < 0.001 vs. AngII 72 h. Treatments with AngII for 72 h are denoted below each bar with a plus sign (+). Losartan was co-treated with AngII for 72 h.

Results showed that MES-13 cells derived from mesangial cells presented low Etd^+^ uptake (Figure 1), and treatment with AngII (10^−7^ M) for different times (0, 24, 48 and 72 h) progressively increased Etd^+^ fluorescence intensities or Etd^+^ uptake rates (Figure 1A–C). At 72 h post AngII application, the Etd^+^ uptake rate increased ~10-fold (from 2.9 ± 1.73 to 32.5 ± 1.30 AU/min). MES-13 cells treated for 72 h with AngII plus Losartan (20 μM)—an AT1 receptor blocker [29]—showed an Etd^+^ uptake rate (3.0 ± 0.2 AU/min) similar to that of cells under control conditions (0 h, 2.9 ± 0.8 AU/min) (Figure 1C). It was also observed that the presence (2.7 ± 0.3 AU/min) or absence (3.2 ± 1.0 AU/min) of fetal bovine serum (FBS) in culture media did not significantly affect Etd^+^ uptake rates (Figure 1C) indicating that FBS does not have an effective concentration of AngII or AngII-like compounds.

### 2.2. AngII Promotes Phosphorylation of MYPT and Increases the Amount of Cx43, Panx1 and P2X_7_R in Mesangial Cells

AngII binding to AT1 receptor activates RhoA and Rho kinase (ROCK) [3], and Cx43 HCs can mediate changes in membrane permeability in different cells types [12,30], we decided to evaluate the activity of RhoA/ROCK and Cx43 HCs. To this end, we first measured the amount of phosphorylated MYPT—a downstream effector of the RhoA/ROCK pathway—and the relative amount of unphosphorylated Cx43 in MES-13 cells at different time periods after treatment with AngII (10^−7^ M). Moreover, and since open Panx1 Chs and P2X_7_Rs could increase membrane permeability and both are co-expressed in several cell types undergoing inflammatory responses [11,31], we also evaluated the relative amount of Panx1 and P2X_7_R.

**Figure 2:**
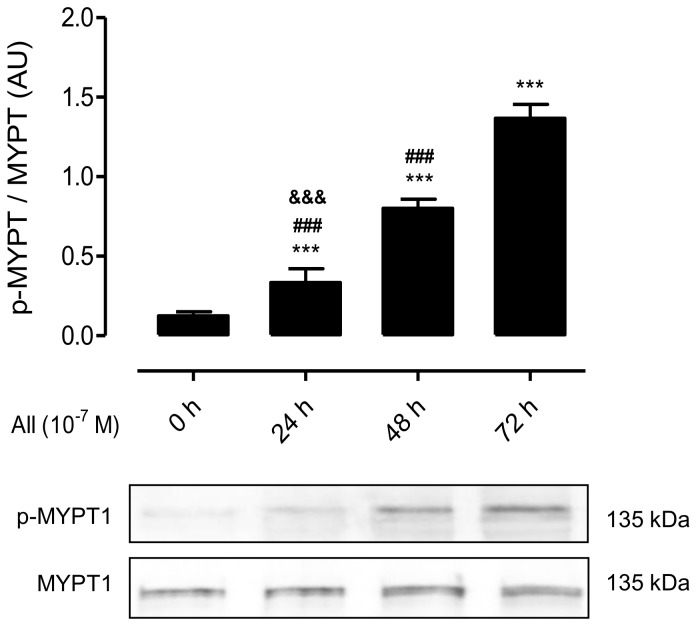
AngII increases phosphorylation of MYPT1 in MES-13 cells. Graphs showing the phosphorylation of MYPT1 evaluated by western blot analysis in MES-13 cells exposed to AngII (10^−7^ M) for different times (0, 24, 48 and 72 h). Each bar represents the mean value ± SE of 4 independent experiments. Statistical significance *** *p* < 0.001 vs. 0 h; ^###^ *p* < 0.001 vs. AngII 72 h; ^&&&^ *p* < 0.001 vs. AngII 48 h. Under the graph are shown representative pictures of p-MYPT and MYPT positive bands and the loading control (α-tubulin).

**Figure 3:**
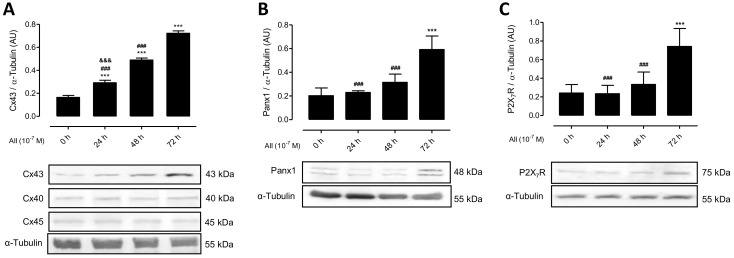
AngII increases the amount of Cx43, Panx1 and P2X_7_R in MES-13 cells. Graphs showing the relative amount of different Cxs (Cx40, Cx43 and Cx45 detected as single bands) (**A**), Panx1 detected as a doublet (**B**) and P2X_7_R detected as single band (**C**) present in the kidney evaluated by western blot analysis in mesangial cells after different times (0 h, 24 h, 48 h and 72 h; Black bars) of treatment with AngII (10^−7^ M). Each bar represents the mean value ± SE of 4 independent experiments. α-tubulin was used as loading control. Statistical significance *** *p* < 0.001 vs. 0 h; ^###^ *p* < 0.001 vs. AngII 72 h; ^&&&^ *p* < 0.001 vs. AngII 48 h. Under the graph are shown representative pictures of Cx43, Cx40, Cx45, Panx1 and P2X_7_R positive bands and loading control (α-tubulin).

Following AngII treatment, the amount of phosphorylated MYPT detected in MES-13 cells was significantly increased at 24 h (From 0.15 ± 0.03 AU to 0.28 ± 0.09 AU), and increased even more at 48 h (0.65 ± 0.16 AU) and 72 h (1.2 ± 0.2 AU) (Figure 2). Similarly, Cx43 was detected as a single band and its amount increased significantly and progressively at 24, 48 and 72 h of stimulation with AngII (From 0.16 ± 0.02 AU at 0 h to 0.30 ± 0.02 AU at 24 h, 0.49 ± 0.02 AU at 48 h and 0.70 ± 0.02 AU at 72 h) (Figure 3A). Since mesangial cells also express Cx40 and Cx45 [32], we evaluated their presence in MES-13 cells. As expected, these two Cxs were detected, but their relative amounts were not affected after treatment with AngII (Figure 3A). This suggests that the effect of AngII could be Cx43-specific. Similarly, the relative amount of Panx1 and P2X_7_R were not significantly different at 24 and 48 h, but were significantly increased at 72 h post-AngII treatment (Panx1 from 0.20 ± 0.03 AU at 0 h to 0.60 ± 0.06 at 72 h and P2X_7_R from 0.24 ± 0.04 AU at 0 h to 0.74 ± 0.10 AU at 72 h) (Figure 3B,C).

### 2.3. AngII-Induced Cell Membrane Permeability is Prevented by the Inhibition of a RhoA/ROCK-Dependent Pathway, and Is Mediated by the Activation of Cx43 HCs, Panx1 Chs and P2X_7_Rs

**Figure 4:**
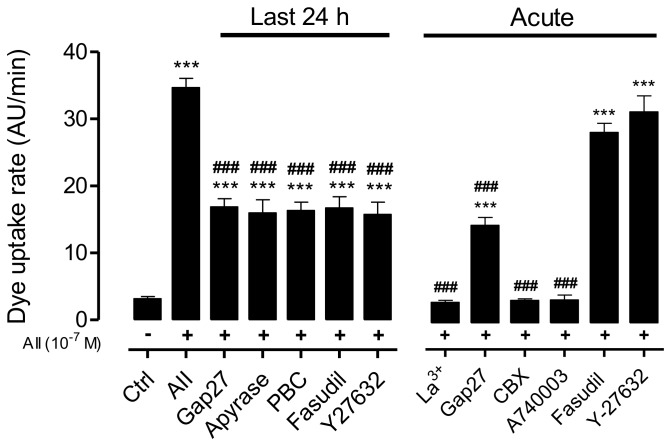
ROCK, Cx43 HC, Panx1 Ch and P2X_7_R blockers reduce the AngII-induced cell membrane permeabilization in MES-13 cells. Quantification of the Etd^+^ uptake rate in MES-13 cells under control conditions, exposed to AngII (10^−7^ M) for 72 h with or without different blockers: Fasudil (15 μM) and Y-27632 (15 μM), ROCK Inhibitors; apyrase (2 units/mL), ATP hydrolase; probenecid (PBC, 500 µM), Panx1 Ch blocker. Lanthanum ion (La^3+^, 200 μM), Cx43 HC and P2X_7_R blocker; the mimetic peptide Gap27 (100 µM), a selective Cx43 HCblocker; carbenoxolone (CBX 10 μM), a Panx1 Ch blocker; A740003 (100 μM), a selective P2X_7_R blocker. Each bar represents the mean value ± SE of ≥3 independent experiments. Statistical significance *** *p* < 0.001 vs. control; ^###^ *p* < 0.001 vs. AngII. Gap27, apyrase, PBC, Fasudil and Y-27632, were added during the last 24 h of treatment with AngII. Acutely were added Gap27 (added 15 min before each Etd^+^ uptake measurement), La^3+^, CBX, A740003, fasudil and Y27632 (each was added 10 min after Etd^+^ uptake). Each treatment with AngII for 72 h is denoted below each bar with a plus sign (+).

To assess whether the AngII-induced increase in cell membrane permeability was mediated by a RhoA/ROCK-dependent pathway, we evaluated the effect of Fasudil and Y-27632—two selective inhibitors of ROCK [3,9]—on AngII-induced Etd^+^ uptake in MES-13 cells. After 72 h, AngII-treated cells showed a significantly higher dye uptake rate compared to that of cells under control conditions (AngII: 34.7 ± 1.34 AU/min; control: 3.16 ± 0.34) (Figure 4). However, the dye uptake rate induced by AngII (72 h) was drastically decreased (~50%) by the mimetic peptide Gap27 (100 µM, 16.9 ± 1.20 AU/min)—a selective Cx43/Cx37 HC blocker [33,34]—as well as apyrase (2 units/mL, 16.3 ± 1.04 AU/min) or probenecid (PBC, 500 µM; 3.5 ± 0.6 AU/min)—a blocker of Panx1 Ch and P2X_7_R [35,36] applied 24 h before completing 72 h of AngII treatment (Figure 4). This suggests that such blockers prevent the progression, but do not reverse the effect on membrane permeability already regenerated by AngII. On the other hand, cells treated with AngII for 72 h and treated with Fasudil (15 µM) or Y-27632 (15 µM) 24 h before evaluating membrane permeability showed increase in Etd^+^ uptake rate that was ~50% lower than those cells treated only with AngII (AngII + Fasudil: 15.4 ± 1.10 AU/min; AngII + Y-27632: 15.8 ± 1.8 AU/min) (Figure 4). This suggests the persistent involvement of membrane pathways found downstream of ROCK. Moreover, since greater Etd^+^ uptake rates in cells treated with AngII for 48 h (11.4 ± 1.70 AU/min) (Figure 1) were like those of cells treated with AngII for 72 h plus Fasudil or Y27632 during the last 24 h, it is possible that ROCK inhibition during the last 24 h of AngII treatment prevented the progression, but did not reverse the downstream-induced increase in membrane permeability already triggered by AngII.

To further identify which non-selective membrane channels are involved in AngII-induced increases in membrane permeability, the acute effects of a set of selective blockers of Cx43 HCs, Panx1 Chs and P2X_7_Rs were also tested. We found a drastic reduction in Etd^+^ uptake rates in cells treated for 72 h with AngII, followed by 15 min treatment with La^3+^ (200 µM)—a known Cx HC and P2X_7_R blocker [37,38]—as well as carbenoxolone (CBX, 10 μM)—a Panx1 Ch inhibitor—or A740003 (100 µM)—a P2X_7_R blocker (Figure 4). Uptake rate values in cells treated with La^3+^ (3.50 ± 0.33 AU/min), CBX (3.80 ± 0.30 AU/min) or A740003 (3.90 ± 0.80 AU/min) were like cells under basal conditions (3.20 ± 0.30 AU/min). In contrast, a significant but not complete decrease in fluorescence intensity was observed (16.5 ± 1.30 AU/min) in MES-13 cells treated acutely with the mimetic peptide Gap27 (100 µM), which is a selective Cx43/Cx37 HC blocker [33,34], suggesting the prevalence of other membrane pathways. Moreover, Etd^+^ uptake rates of cells treated acutely with Fasudil or Y-27632 were about the same as those of cells treated with AngII for 72 h (Figure 4), suggesting that these two compounds are not inhibitors of Cx HCs, Panx1 Chs or P2X_7_R, which are involved in the increase in membrane permeability induced by AngII.

### 2.4. AngII Reduces Intercellular Communication Mediated by GJs in MES-13 Cells

**Figure 5:**
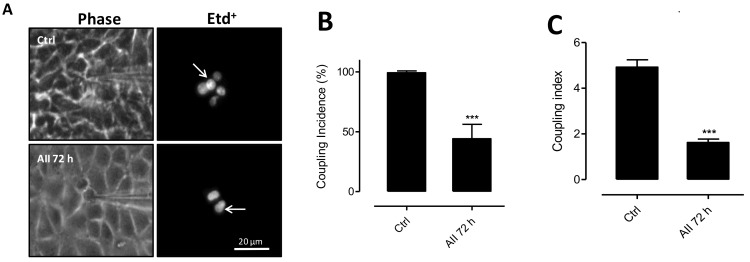
AngII decreases gap junctional coupling between mesangial cells. (**A**) Etd^+^ was microinjected into the brightest cell (arrow) and diffused to neighboring cells. The right panels show fluorescence of Etd^+^ at different times (0 and 72 h) and the left panels are the phase contrast images. Coupling incidence (**B**) and coupling index (**C**) were evaluated in confluent mesangial cell cultures with AngII for different time periods (0 and 72 h) using dye (Etd^+^, 25 μM) coupling technique (black bars). Each bar represents the mean value ± SE of 4 independent experiments. In each experiment the dye was microinjected into at least 10 cells. Statistical significance *** *p* < 0.001 vs. Control. Scale bar = 20 μm.

Since increases in membrane permeability via HCs induced by various conditions also reduces intercellular communication mediated by GJs [15,16,39], we decided to evaluate the effect of AngII on GJs expressed by MES-13 cells. The functional state of GJs can be demonstrated by means of an intercellular transfer of dyes microinjected in single cells of clusters or monolayers [12]. With this experimental approach, control MES-13 cells were found to be dye-coupled (Figure 5A), and the coupling incidence decreased (44 ± 6%) after 72 h treatment with AngII (10^−7^ M) compared to control cells (99 ± 1%) (Figure 5A). In addition, the number of coupled cells (coupling index) also decreased from 5.0 ± 0.2 cells under control conditions to 2.0 ± 0.1 cells after 72 h treatment with AngII (Figure 5B,C), hence indicates that AngII impacts membrane permeability and GJ channels in an opposite manner.

**Figure 6:**
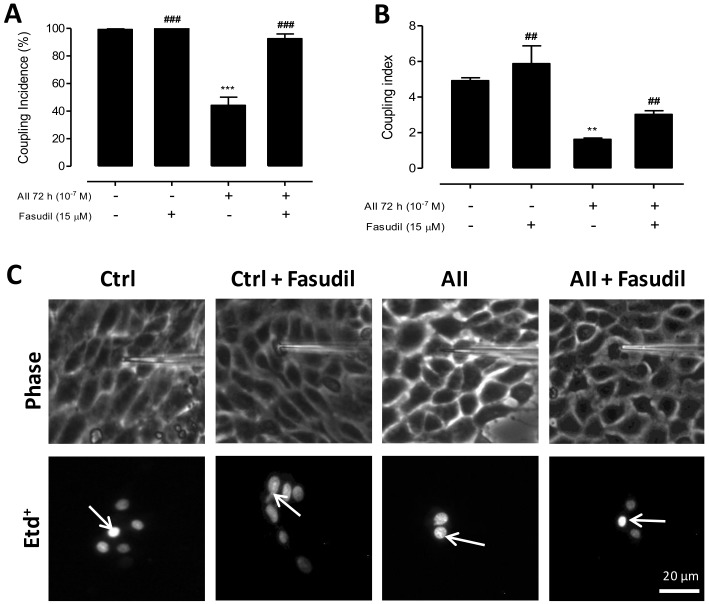
Fasudil increases gap junctional coupling between mesangial cells treated with AngII for 72 h. Coupling incidence (**A**) and coupling index (**B**) were evaluated in confluent mesangial cells (black bars) using a dye (Etd^+^, 25 μM) coupling technique, under control conditions or exposed at AngII (10^−7^ M) for 72 h. Fasudil (15 µM) was added during the last 24 h of incubation with AngII. Each treatment is denoted below each bar with a plus sign (+). Each bar represents the mean value ± SE of 4 independent experiments. In each experiment the dye was microinjected into at least 10 cells. Statistical significance ** *p* < 0.01 and *** *p* < 0.001 vs. Ctrl; ^##^ *p* < 0.001 and ^###^ *p* < 0.001 vs. AngII. (**C**) Etd^+^ was microinjected into the brightest cell (arrow) and diffused to neighboring cells. Top panels are the phase contrast images and bottom panels show Etd^+^ fluorescence in cells under different treatments. Scale bar = 20 μm.

We also evaluated the effect of Fasudil (15 µM) on the functional state of GJs in AngII-treated MES-13 cells. After 72 h treatment with AngII (10^−7^ M), cells showed a reduced coupling incidence (44 ± 6%) compared to control cells (control, 99.0 ± 0.8% and control + Fasudil, 100 ± 0%), but Fasudil added the last 24 h to cells treated with AngII reduced the inhibitory effect of AngII on intercellular communication via GJs (incidence of coupling: 93.0 ± 3.4%) (Figure 6A). In addition, the coupling index was also decreased (1.0 ± 0.8 cells) in MES-13 cells treated with AngII for 72 h compared to control cells (control: 5.0 ± 0.2; control + Fasudil: 6.0 ± 1.0 cells). However, cells treated with AngII for 72 h and Fasudil during the last 24 h showed a significantly less reduction in the number of coupled cells (3.0 ± 0.2 cells) (Figure 6B,C) as compared to cells treated only with AngII (1.0 ± 0.8 cells).

### 2.5. Inhibition of RhoA/ROCK Reduces the Amount of Phosphorylated MYPT and Cx43, but Does Not Impact the Amount of Panx1 or P2X_7_ Receptors in AngII-Treated MES-13 Cells

**Figure 7:**
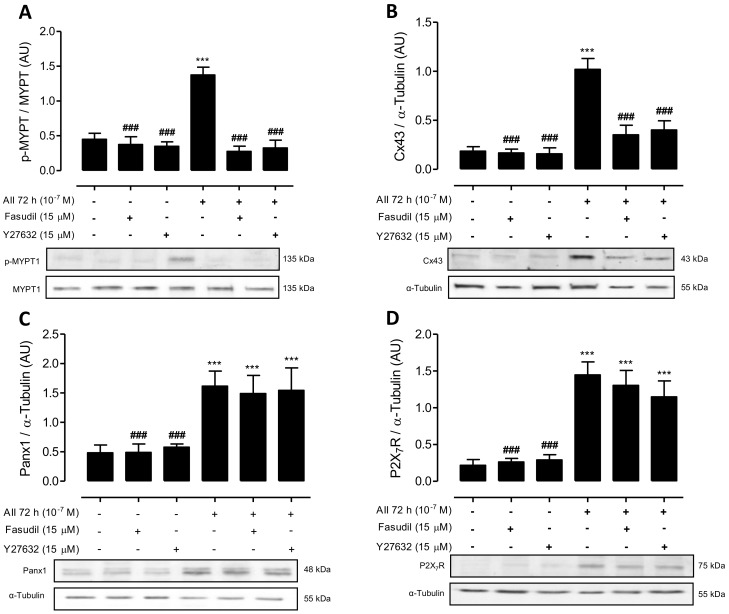
Fasudil or Y-27632 decreases the amount of phosphorylated MYPT and Cx43 without affecting the amount of Panx1 and P2X_7_R in MES-13 cells treated with AngII. Graphs show phosphorylation of MYPT-1 (**A**), the relative amount of Cx43 (**B**), Panx1 (**C**) and P2X_7_R (**D**) determined by western blot analysis in MES-13 cells under control conditions or exposed to AngII (10^−7^ M) for 72 h. Fasudil (15 µM) or Y-27632 (15 µM) were added 24 h before harvesting the cells. Each treatment is denoted below each bar with a plus sign (+). Each bar represents the mean value ± SE of 4 independent experiments. Statistical significance *** *p* < 0.001 vs. Ctrl; ^###^ *p* < 0.001 vs. AngII. Under the graph representative pictures of Cx43, phosphorylated MYPT (p-MYPT) and unphosphorylated MYPT positive bands and its loading control (α-tubulin) are shown.

An increase in phosphorylation of MYPT was detected in MES-13 cells after 72 h of AngII stimulation (1.4 ± 0.1 AU) compared to control cells (control 0.45 ± 0.09 AU) or control cells treated with Fasudil (0.38 ± 0.10 AU) or Y-27632 (0.35 ± 0.06 AU) (Figure 7A). This suggests that Rho/ROCK activity is very low under control conditions. Moreover, we found that Fasudil and Y-27632 added the last 24 h to cell cultures pretreated with AngII for 72 h reduced the amount of phosphorylated MYPT (AngII + Fasudil, 0.28 ± 0.08 AU and AngII + Y-27632, 0.33 ± 0.10 AU), reaching a phosphorylation state as low as that of control cells (Figure 7A).

We also evaluated the effect of Fasudil (15 µM) and Y-27632 (15 µM) on the amount of Cx43, Panx1 and P2X_7_R in MES-13 cells treated with AngII (10^−7^ M). An evident increase in the relative amount of Cx43 was found in MES-13 cells after 72 h treatment with AngII (1.0 ± 0.1 AU) compared to control cells (0.18 ± 0.04 AU), or control cells treated with Fasudil (0.17 ± 0.04 AU) or Y-27632 (0.16 ± 0.06 AU). But, Fasudil or Y-27632 added the last 24 h to MES-13 cells pretreated with AngII for 72 h drastically prevented a rise in Cx43 induced by AngII (AngII + Fasudil, 0.45 ± 0.10 AU and AngII + Y-27632, 0.4 ± 0.1 AU), reaching values close to those found in control cells (Figure 7B). In contrast, Rho/ROCK inhibition during the last 24 h of AngII treatment did not significantly affect the amount of Panx1 or P2X_7_R, which were comparable to those of cells treated only with AngII (Figure 7C,D). Therefore, it can be inferred that increases in phosphorylated MYPT and Cx43 are probably controlled by the same RhoA/ROCK-dependent pathway, and the amounts of Panx1 and P2X_7_R are controlled by a mechanism independent of RhoA/ROCK.

### 2.6. Inhibition of Panx1 Chs, Cx43 HCs, or P2X_7_Rs Does Not Affect AngII-Induced RhoA/ROCK Activation in MES-13 Cells

**Figure 8:**
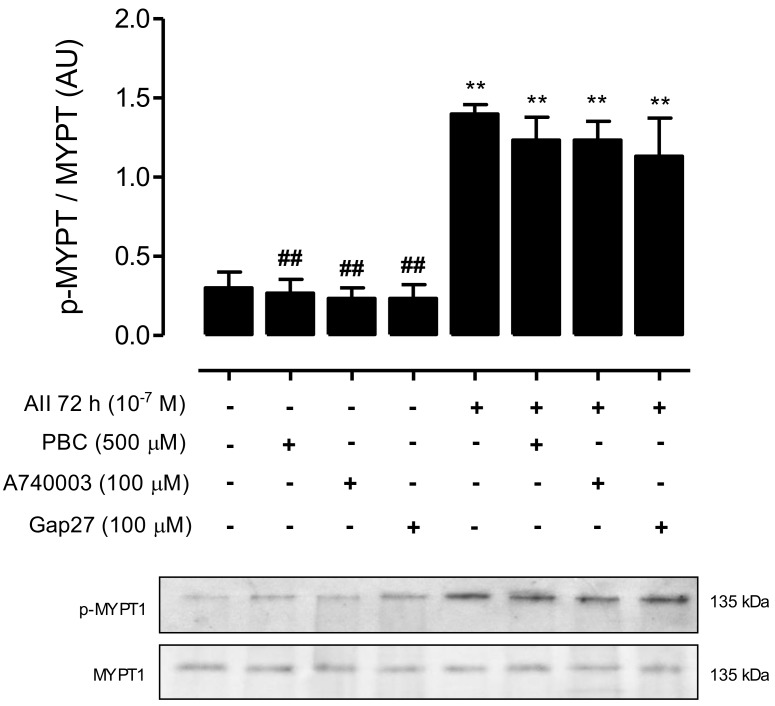
Blockade of Cx43 HCs, Panx1 Chs or P2X_7_Rs does not affect the amount of phosphorylated MYPT in AngII-treated MES-13 cells. Graph showing amount of phosphorylated MYPT detected by western blot assays in MES-13 cells under control conditions, exposed to AngII (10^−7^ M) for 72 h or exposed to AngII for 72 h with different blockers: probenecid (PBC, 500 µM), Panx1 Ch blocker; A740003 (100 μM), a selective P2X_7_R blocker; and the mimetic peptide Gap27 (100 µM), a selective Cx43 HC blocker. Each bar represents the mean value ± SE of 3 independent experiments. Statistical significance ** *p* < 0.01 vs. Ctrl; ^##^ *p* < 0.01 vs. AngII. Under the graph representative pictures of p-MYPT and MYPT positive bands are shown. PBC, A740003 and Gap27 were co-added for 72 h with AngII. Each treatment is denoted below each bar with a plus sign (+).

To elucidate whether Cx HCs, Panx1 Chs and P2X_7_Rs participate in AngII-induced RhoA/ROCK activation, we evaluated the effects of selective blockers for each channel type—Gap27, PBC and A740003, respectively—applied during the last 24 h on the amount of phosphorylated MYPT in MES-13 cells treated with AngII (10^−7^ M) for 72 h. First, we found that these channel blockers applied for 24 h to cells under control conditions did not alter the amount of phosphorylated MYPT (control, 0.30 ± 0.10 AU; control + PBC, 0.17 ± 0.04 AU; control + Gap27, 0.23 ± 0.09 AU; and control + A740003, 0.16 ± 0.06 AU) (Figure 8). Moreover, the amount of phosphorylated MYPT in cells treated with AngII for 72 h (1.4 ± 0.06 AU) was not significantly affected by PBC (1.2 ± 0.12 AU), Gap27 (1.2 ± 0.15 AU) or A740003 (1.1 ± 0.24 AU) (Figure 8). Therefore, RhoA/ROCK activation by AngII does not require functional Panx1 Chs, Cx HCs or P2X_7_Rs, indicating that activation of these channels occurs downstream of the AngII-activated pathway.

### 2.7. Inhibition of RhoA/ROCK, Cx HCs or P2X_7_Rs, but Not Panx1 Chs, Prevents Increases in Lipid Peroxidation and Inflammatory Responses Induced by AngII in MES-13 Cells

**Figure 9:**
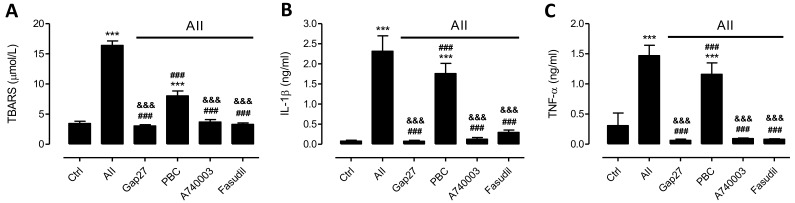
Blockade of ROCK, Cx HCs or P2X_7_Rs decreases the amount of TBARS, TNF-α and IL-1β in AngII-treated MES-13 cells. Graphs showing amount of (**A**) TBARS, (**B**) IL-1β and (**C**) TNF-α in the culture medium of MES-13 cells under control conditions or after 72 h exposure to AngII (10^−7^ M) in the presence of different blockers: Gap27 (100 µM), a selective Cx43 HC blocker; probenecid (PBC, 500 µM), Panx1 Ch blocker; A740003 (100 μM), a selective P2X_7_R blocker or Fasudil (15 μM), ROCK inhibitor applied 24 h before harvesting the medium samples. Each bar represents the mean value ± SE of 4 independent experiments. Statistical significance, *** *p* < 0.001 vs. Ctrl; ^###^ *p* < 0.001 vs. AngII; ^&&&^ *p* < 0.001 vs. AngII + PBC.

Since AngII is known to induce Ca^2+^ influx as well as Ca^2+^ release from intracellular stores [40,41] leading to oxygen radical generation and cell damage in several kidney diseases [5,42], and P2X_7_Rs as well as some Cx HCs are Ca^2+^ conductive channels [43,44], we decided to evaluate whether AngII treatment causes OS and pro-inflammatory cytokine production in mesangial cells. Here, we show that the extracellular amount of TBARS increased (16.40 ± 0.70 µmol/L) in MES-13 cells treated with AngII (10^−7^ M) for 72 h compared to control conditions (3.40 ± 0.40 µmol/L) (Figure 9A). It was also found that this AngII treatment increased the extracellular amount of IL-1β and TNF-α (IL-1β, 2.30 ± 0.40 ng/mL; TNF-α, 1.50 ± 0.20 ng/mL) compared to control conditions (IL-1β, 0.08 ± 0.02 ng/mL; TNF-α, 0.31 ± 0.21 ng/mL) (Figure 9B). Moreover, treatment with Fasudil at 24 h prior to the end of the 72 h incubation with AngII significantly reduced the extracellular amount of TBARS (3.31 ± 0.23 µmol/L) as well as IL-1β and TNF-α (IL-1β, 0.30 ± 0.06 ng/mL; TNF-α, 0.08 ± 0.01 ng/mL), as compared to what was found in the extracellular medium of cells treated only with AngII, and were similar to those found in the extracellular space of control cell cultures (Figure 9).

Interestingly, treatment with Gap27 or A740003 24 h prior to the end of the 72 h post-treatment with AngII significantly reduced the amount of TBARS (Gap27: 3.04 ± 0.22 µmol/L and A740003: 3.70 ± 0.40 µmol/L). However, treatment with PBC caused only a partial reduction in the amount of TBARS (8.03 ± 0.82 µmol/L) (Figure 9A). Moreover, the extracellular amount of IL-1β and TNF-α in MES-13 cell cultures treated with AngII for 72 h was more than 10 times higher than that of control cultures (Figure 9B,C). However, the amount of pro-inflammatory cytokines in cultures treated with AngII for 72 h and then exposed to Gap27 (IL-1β, 0.08 ± 0.02 ng/mL and TNF-α, 0.06 ± 0.02 ng/mL) or A740003 (IL-1β, 0.13 ± 0.04 ng/mL and TNF-α, 0.09 ± 0.01 ng/mL) 24 h before harvesting the extracellular solution was comparable to that of cell cultures under control conditions (Figure 9B,C). Unexpectedly, in cultures treated with AngII plus PBC the amount of pro-inflammatory cytokines was slightly, but not significantly, lower as compared to cultures treated only with AngII (IL-1β, 1.76 ± 0.25 ng/mL and TNF-α, 1.16 ± 0.19 ng/mL) (Figure 9B,C).

## 3. Discussion

**Figure 10:**
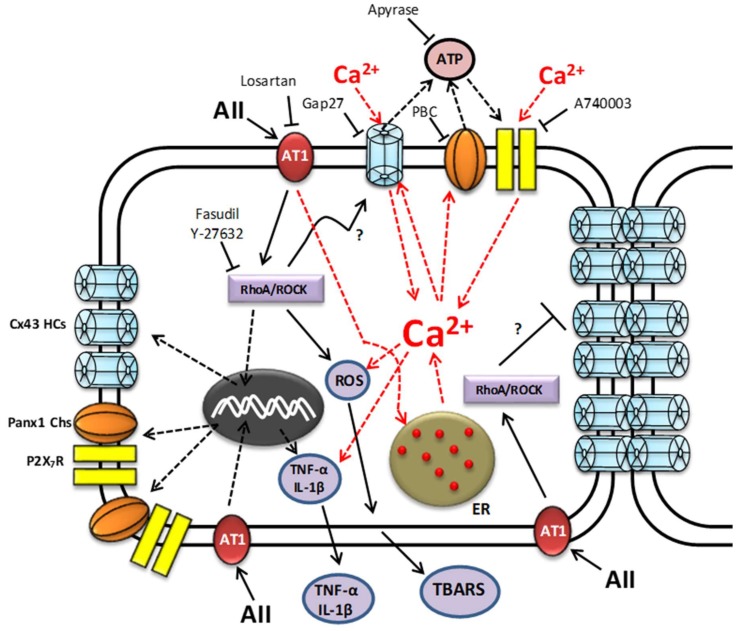
Scheme of possible signaling pathways involved in regulating the functional state of Cx43 HCs, Panx1 Chs, P2X_7_Rs and Cx GJs in mesangial cells stimulated with AngII. High AngII concentrations (continuous black arrows) could promote Ca^2+^ release from the endoplasmic reticulum (ER) and activation of a RhoA/ROCK-dependent pathway through angiotensin type 1 receptors (AT1R). The latter is inhibited by losartan. Once the RhoA/ROCK-dependent pathway is activated, the expression and release of pro-inflammatory cytokines such as TNF-α and IL-1β as well as formation of reactive oxygen species (ROS) that generate thiobarbituric acid reactive substances (TBARS) upon reaction with lipid of cell membranes can occur. The activated RhoA/ROCK-dependent pathway could affect the function of Cx43 HCs by opening them, since Fasudil or Y-27632 (ROCK blockers) inhibit this response. The resulting increase in intracellular Ca^2+^ can also activate Panx1 Chs and together with activated Cx HCs enable release of ATP to the extracellular medium. Then, the extracellular ATP activates P2X_7_ receptors that together with Cx43 HCs permit a drastic increase in Ca^2+^ influx. The resulting intracellular Ca^2+^ concentration, as already seen in other systems, promote the expression and release of pro-inflammatory cytokines such as TNF-α, IL-1β, and the generation of ROS. The release of ATP and influx of Ca^2+^ establish a positive feedback loop. This loop is inhibited by different compounds: Apyrase, ATP hydrolase; the mimetic peptide Gap27, a selective Cx43 HCs blocker; A740003, a selective P2X_7_R blocker or probenecid (PBC), an inhibitor of Panx1 Chs. This increase in the cellular activity caused by AngII, where the RhoA/ROCK pathway could be involved, also reduces cell–cell communication through GJs, further affecting the cellular integrity. Discontinuous red and black arrows indicate cell responses identified in other systems, whereas continuous black arrows denote responses identified in the present work.

In the present work, high levels of TBARs, IL-1β and TNF-α were detected in the extracellular milieu of AngII-treated MES-13 cells, which are characteristic features of an inflammatory response. This outcome was preceded by an increase in membrane permeability due to the activation of at least three non-selective membrane channels (Cx HCs, Panx1 Ch and P2X_7_R) mainly through a RhoA/ROCK-dependent intracellular signaling pathway promoted by the activation of AT1 receptors. The generation of TBARS and pro-inflammatory cytokines was strongly dependent on the upregulation of functional Cx HCs as well as P2X_7_Rs, and to a lesser but still significant extent on Panx1 Chs (Figure 10). Thus, we propose that in addition to AT1 receptor and RhoA/ROCK inhibition, AngII-induced mesangial cell damage could be efficiently prevented by inhibiting Cx43 HCs or P2X_7_Rs.

The AT1 receptor activated by AngII is a key factor in the pathogenesis of renal damage, since it triggers several intracellular signaling cascades, such as a RhoA/ROCK-dependent pathway. The latter contributes to inflammatory and oxidative changes observed in renal diseases [3,6], but the mechanism is not completely understood. InAngII-treatedMES-13 cells, we found an AT1 receptor-dependent increase in RhoA/ROCK activity reflected by the progressive increase in the amount of phosphorylated MYPT. This conclusion is supported by our finding that Losartan—an inhibitor of AT1 receptors—prevented the AngII-induced increase in membrane permeability. Furthermore, ROCK inhibition with Fasudil or Y-27632 prevented the AngII-induced increase in the amount of phosphorylated MYPT (Figure 10).

The half-life of bioactive peptides such as AngII is likely to be about a few hours [45], and the effect of AngII on membrane permeability described here was significant 2–3 days after its application, suggesting that its effects on MES-13 cells resulted from slow metabolic responses rather than the direct activation of channels present at the cell membrane of control cells. However, soon after AngII binds to AT1 occurs the increase in cytoplasmic free Ca^2+^ concentration [40,41] as well as activation of Akt [46], and these responses might be sufficient to increase the open probability of Cx HCs [47] and Panx1 CHs [48,49]. Interestingly, these two channels are permeable to ATP, and could enable the release of this purine to the extracellular milieu, thus favoring the activation of P2X_7_Rs also expressed in control MES-13 cells. Since P2X_7_Rs and Cx43 HCs are permeable to Ca^2+^ [19,43], it is possible that the concentration of AngII used in the present work caused a persistent increase in intracellular free Ca^2+^ concentration, contributing to maintaining the activity of Cx HCs and Panx1 Chs available at the cell membrane (Figure 10). In favor of this interpretation, we recorded high membrane permeability to Etd^+^ 48 and 72 h after AngII treatment in cells bathed in AngII-free saline solution. A possible explanation for this delayed response could be that a calcium code triggered by AngII either enhanced the expression and/or reduced the degradation of proteins such as Cx43, Panx1 and P2X_7_R. Interestingly, the de novo expression of Cx HCs and P2X_7_Rs in denervated muscles has been shown to increase the activity of proteolytic pathways, which lead to muscle atrophy [50]. Thus, our data, showing a progressive increase in the amount of Cx43 caused by AngII, may have resulted from an increase in gene expression (Figure 10) rather than a reduction in protein degradation. Increases in Cx43 and Panx1 detected by western blot suggest that progressively more channels were available at the cell surface. Therefore, greater membrane permeability to Etd^+^ in MES-13 cells induced by AngII could be explained by two responses: (1) increases in amounts of Cx43, Panx1 and P2X_7_R; and (2) increases in the open probability of Cx43 HCs and Panx1 Chs. In favor of the role played by Cx HCs and Panx1 Chs in increasing AngII-induced membrane permeability is the fact that these two channels are permeable to Etd^+^ [28]. Additionally, greater permeability was abrogated by selective blockers of these channels (Gap27 for Cx43 HCs, and PBC and low CBX concentrations for Panx1 Chs) (Figure 10). Possible implications of these findings could be relevant to explaining increases in the amount of Cx43 protein, mainly in mesangial cells found by others in at least three models of renal injury [10,51]. Interestingly, we found similar changes in RhoA/ROCK activity and ROCK inhibitors, which prevented increases in amount of Cx43 induced by AngII, indicating that expression and activation of RhoA/ROCK and Cx43 are regulated by the same membrane transduction mechanism and intracellular signaling pathway activated by AngII (Figure 10). This interpretation is also supported by the strong reduction of the AngII dependent increase in the amounts of phosphorylated MYPT and Cx43 caused by inhibitors of ROCK (Fasudil applied in the last 24 h of the AngII treatment) (Figure 10). The same metabolic pathway seems to be involved in the reduction of cell-cell communication mediated by GJs between MES-13 cells, since inhibition of ROCK significantly reduced the cell-cell uncoupling induced by AngII (Figure 10). Both the activity of a RhoA/ROCK-dependent pathway and the amount of Cx43 has been observed to increase in various models of renal damage [10,24,25,52]. Interestingly, a comparable response has been observed in corneal epithelial cells, where a RhoA/ROCK-dependent pathway is involved in the formation of Cx43 GJs. In fact, ROCK inhibition resulted in greater cell-cell communication mediated by Cx43 GJs [53]. A similar relationship between the RhoA/ROCK pathway and Cx43 has been observed in fibroblasts, where fibroblast expansion in response to tissue stretch involves extracellular ATP signaling through the RhoA/ROCK pathway and the activity of Cx HCs. In this system, Y-27632 or Cx HC blockers (Octanol and CBX) prevent increases in extracellular ATP concentration and in fibroblast expansion [54]. In agreement with this mechanism, Cx HCs and Panx1 Chs enable the release of ATP to the extracellular milieu [35,55]. Despite the direct relationship between the RhoA/ROCK pathway and Cx43 postulated in the previous work mentioned above, in our work some remaining membrane permeability was observed in mesangial cells stimulated with AngII and treated with the mimetic peptide Gap27 (Cx43 HC blocker) as well as ROCK inhibitors (Fasudil and Y-27632). These results could be explained by the involvement of other membrane channels such as Panx Chs.

The involvement of extracellular ATP in the AngII-induced effects on MES-13 cells was evident, since AngII-induced membrane permeability was drastically diminished upon exposure to: (1) apyrase that degrades ATP; (2) A740003, which is a selective inhibitor of P2X_7_Rs activated by extracellular ATP; and (3) PBC or low concentration of CBX—inhibitors of Panx1 Chs—and Gap27, inhibitor of Cx43 HCs, which are blockers of two membrane pathways through which ATP can be released to the extracellular milieu (Figure 10).

The AngII-induced generation of OS, IL-1β and TNF-α could explain decreases in intercellular communication mediated by GJs, since reduced redox potential and pro-inflammatory cytokines have also been shown to cause such effect in cortical astrocytes and in endothelial cells of the blood-brain barrier [15,30,56,57,58]. Accordingly, Fasudil or Y-27632 has been shown to decrease AngII-induced inflammation and OS in models of kidney diseases [1,42]. For example, ROCK inhibitors protect rats subjected to chronic AngII infusion from translocation of p65 to the nuclei of cells from the afferent arterioles, suggesting that a RhoA/ROCK-dependent pathway is involved in NF-κB activation, and the ROCK/NF-κB axis contributes to the AngII-induced upregulation of angiotensinogen [59]. Additionally, Fasudil has a renoprotective action in spontaneous hypertensive rats under deoxycorticosterone-acetate salt treatment [60].

The participation of Cx HCs, Panx1 Chs and P2X_7_Rs in different tissues undergoing inflammation has been demonstrated, and the inhibition or ablation of Cx HCs has been shown to confer significant tissue protection [16,37,50,61,62,63]. In MES-13 and primary mesangial cells, AngII seems to promote a similar feedforward mechanism in which these three non-selective channels, without excluding others (Figure 10) (i.e., TRPC6 channels; [64]), maintain or even amplify inflammatory and oxidative responses, causing damage to kidney cells. Therefore, we propose that blocking AngII-induced progression in mesangial cell damage could be accomplished by inhibiting the RhoA/ROCK as previously demonstrated. Moreover, the effective reduction of initial AngII-induced alterations in cell membrane permeability-leading to activation of several metabolic pathways that promote OS and generation of pro-inflammatory cytokines—can be accomplished with selective and potent inhibitors of non-selective channels (Figure 10).

## 4. Materials and Methods

### 4.1. Reagents

Angiotensin II (AngII) was obtained from Alomone (Jerusalem, Israel); Fasudil, Y-27632, ethidium (Etd^+^) bromide, lanthanum (La^3+^) chloride, Losartan and malondialdehyde (MDA) were obtained from Sigma-Aldrich (St. Louis, MO, USA); Gap27—a peptide that inhibits Cx43 HCs—was obtained from NeoMPS, SA (Strasbourg, France); A740003—a specific P2X_7_Rblocker—was obtained from Tocris Biosciences (Bristol, UK). The monoclonal anti-α-tubulin antibody was obtained from Sigma-Aldrich (St. Louis, MO, USA); the polyclonal anti-phosphorilated-MYPT1 (Thr696) antibody was obtained from Merck Millipore (Darmstadt, Germany); the monoclonal anti-MYPT1 antibody was obtained from BD Transduction Laboratories (San José, CA, USA); the monoclonal anti-unphosphorylated Cx43 antibody was obtained from Invitrogen (Carlsbad, CA, USA); a polyclonal anti-Panx1 antibody described previously [65] was used; the polyclonal anti-P2X_7_R antibody was obtained from Abcam (Cambridge, UK); anti-mouse and anti-rabbit secondary antibodies conjugated to horseradish peroxidase were from Santa Cruz Biotechnology Inc. (Santa Cruz, CA, USA).

### 4.2. Cell Culture

The cell line MES-13, derived from mesangial cells (CRL-1927 from ATCC, Manassas, VA, USA), was cultured and treated with AngII (10^−7^ M) for different time periods (0, 24, 48 and 72 h) in a 2:1 mixture of DMEM and F-12 tissue culture media supplemented with 100 U/mL penicillin and 100 μg/mL streptomycin. Cells were kept at 37 °C in a 5% CO_2_/95% air atmosphere, at nearly 100% relative humidity. Fasudil (15 μM) or Y-27632 (15 μM) were added during the last 24 h to cell cultures treated with AngII for 72 h.

### 4.3. Dye Uptake

Cx HC activity was evaluated by using the dye uptake method previously published [55]. In brief, cells were plated onto glass coverslips and bathed with Locke’s saline solution (in mM: 154 NaCl, 5.4 KCl, 2.3 CaCl_2_, 1.5 MgCl_2_, 5 HEPES, 5 glucose, and pH 7.4) containing 5 μM Etd^+^, which is a membrane-impermeant cationic dye. In time-lapse experiments images were recorded (at regions of interest in different cells) every 30 s during 13 min using a Nikon Eclipse Ti inverted microscope (Tokio, Japan) and NIS-Elements software. Basal fluorescence signal was recorded in cells only in the presence of Locke’s saline solution that contained divalent cations.

### 4.4. Dye Coupling

MES-13 cells seeded on glass coverslips were bathed with Locke’s saline solution, and cultures were observed using an inverted microscope equipped with a xenon arc lamp and a Nikon B filter (excitation wavelength: 450–490 nm, emission wavelength: above 520 nm). Etd^+^ (25 mM) was microinjected through a glass microelectrode to one cell. Dye transfer to neighboring cells was evaluated two minutes after injection. We routinely performed all dye coupling experiments in the presence of La^3+^ (150 µM) to block the Cx HCs and prevent Etd^+^ leakage through open hemichannels that would reduce the intercellular diffusion among coupled cells [66]. The incidence of coupling corresponded to the percentage of cases in which the dye spread occurred to at least one neighboring cell. The coupling index was calculated as the average number of cells to which the dye had spread, divided by the number of positive cases. Intracellular coupling was tested in all experiments by injecting a minimum of 10 cells.

### 4.5. Western Blot Assays

Cell cultures were placed on ice, washed twice with ice-cold PBS (pH 7.4), harvested by scraping in 80 μL of a solution containing a protease and phosphatase inhibitor cocktail (Thermo Scientific, Pierce, Rockford, IL, USA; cat # 78430). Lysates were centrifuged (25,200× *g*, Eppendorf Centrifuge 5415C, Hamburg, Germany), and supernatants were collected for Western blot analysis. Protein concentration was determined by using the Lowry’s method [67]. Samples of homogenized cell cultures (50 μg of proteins) under different conditions were resolved by electrophoresis in 10% SDS-polyacrylamide gel (SDS-PGE), and in one lane pre-stained molecular weight markers contained in an aliquot were resolved. Proteins were transferred to a PVDF membrane (pore size: 0.45 μm), which was blocked at room temperature with Tris pH 7.4, 5% skim milk (*w*/*v*) and 1% BSA (*w*/*v*). Then, the PVDF membrane was incubated overnight at 4 °C with anti-Cx43 (1:1000), anti-p-MYPT1 (1:500), anti-MYPT1 (1:1000), anti-Panx1 (1:1000) or anti-P2X_7_R (1:1000) antibody, followed by incubation with rabbit or mouse secondary antibody conjugated to peroxidase (1:2000 both of them) for 1 h at room temperature. Then, the PVDF membrane was stripped and reblotted with the anti-α-tubulin antibody (1:5000) used as loading control, following the same procedure described above. After repeated rinses, immunoreactive proteins were detected by using ECL reagents (Pierce Biotechnology, Rockford, IL, USA) according to the manufacturer’s instructions. The bands detected were digitized and subjected to densitometry analysis using the software Image J (Version 1.50i, NIH, Washington, DC, USA).

### 4.6. Thiobarbituric Acid Reactive Substances (TBARS) Measurement

The amount of TBARS was estimated using the method described by Ramanathan and collaborators [68] with slight modifications. Culture medium was mixed with SDS (8% *w*/*v*), thiobarbituric acid (0.8% TBA *w*/*v*), and acetic acid (20% *v*/*v*), and heated for 60 min at 90 °C. Precipitated material was removed by centrifugation, and the absorbance of the supernatant was evaluated at 532 nm. The amount of TBARS was calculated using a calibration curve obtained with malondialdehyde (MDA) as standard. MDA was obtained from Merck (Darmstadt, Germany).

### 4.7. Enzyme-Linked Immunosorbent Assay

IL-1β and TNF-α ELISA assays were performed to determine the amount of IL-1β and TNF-α in the extracellular medium under different conditions, following the manufacturer’s protocol (IL-1β and TNF-α EIA kit, Enzo Life Science, Farmingdale, NY, USA). Results were normalized by protein amount in ng/mL.

### 4.8. Statistical Analysis

Results were evaluated by ANOVA, and the Tuckey’s post-test was used to evaluate the difference between the two groups. Results below are expressed as the average of values from each independent experiment ± SE, and considered significantly different if *p* < 0.05. Analyses were performed with the GraphPad Prism 5 software for Windows (1992–2007, GraphPad Software, 12 March, 2007, La Jolla, CA, USA).

## Abbreviations

Cx43	Connexin 43
Panx1	Pannexin 1
P2X_7_R	P2X_7_ Receptor
OS	Oxidative stress
TBARS	Thiobarbituric reactive species
AngII	Angiotensin II
ROCK	Rho kinase
NFκB	Nuclear factor kappa-light-chain-enhancer of activated B cells
Cx GJs	Connexin gap junctions
Cx HCs	Connexin hemichannels
Panx Chs	Pannexin channels
FBS	Fetal bovine serum
CBX	Carbenoxolone
PBC	Probenecid
MDA	Malondialdehyde
Etd^+^	Ethidium

## References

[1] Ozawa Y., Kobori H., Suzaki Y., Navar L.G. (2007). Sustained renal interstitial macrophage infiltration following chronic angiotensin II infusions. Am. J. Physiol. Ren. Physiol..

[2] Border W.A., Noble N.A. (1998). Interactions of transforming growth factor-beta and angiotensin II in renal fibrosis. Hypertension.

[3] Rúperez M., Sánchez-Lopez E., Blanco-Colio L.M., Esteban V., Rodriguez-Vita J., Plaza J.J., Egido J., Ruíz-Ortega M. (2005). The Rho-kinase pathway regulates angiotensin II-induced renal damage. Kidney Int. Suppl..

[4] Guilluy C., Rolli-Derkinderen M., Loufrani L., Bourge A., Henrion D., Sabourin L., Loirand G., Pacaud P. (2008). Ste20-related kinase SLK phosphorylates Ser188 of RhoA to induce vasodilation in response to angiotensin II Type 2 receptor activation. Circ. Res..

[5] Peng F., Wu D., Gao B., Ingram A.J., Zhang B., Chorneyko K., McKenzie R., Krepinsky J.C. (2008). RhoA/Rho-kinase contribute to the pathogenesis of diabetic renal disease. Diabetes.

[6] Kolavennu V., Zeng L., Peng H., Wang Y., Danesh F.R. (2008). Targeting of RhoA/ROCK signaling ameliorates progression of diabetic nephropathy independent of glucose control. Diabetes.

[7] Zhou L., Liu F., Huang X.R., Liu F., Chen H., Chung A.C., Shi J., Wei L., Lan H.Y., Fu P. (2011). Amelioration of albuminuria in ROCK1 knockout mice with streptozotocin-induced diabetic kidney disease. Am. J. Nephrol..

[8] Loirand G. (2015). Rho Kinases in Health and Disease: From Basic Science to Translational Research. Pharmacol. Rev..

[9] Kushiyama T., Oda T., Yamamoto K., Higashi K., Watanabe A., Takechi H., Uchida T., Oshima N., Sakurai Y., Miura S. (2013). Protective effects of Rho kinase inhibitor fasudil on rats with chronic kidney disease. Am. J. Physiol. Ren. Physiol..

[10] Toubas J., Beck S., Pageaud A.L., Huby A.C., Mael-Ainin M., Dussaule J.C., Chatziantoniou C., Chadjichristos C.E. (2011). Alteration of connexin expression is an early signal for chronic kidney disease. Am. J. Physiol. Ren. Physiol..

[11] Silverman W.R., de Rivero Vaccari J.P., Locovei S., Qiu F., Carlsson S.K., Scemes E., Keane R.W., Dahl G. (2009). The pannexin 1 channel activates the inflammasome in neurons and astrocytes. J. Biol. Chem..

[12] Sáez J.C., Berthoud V.M., Brañes M.C., Martínez A.D., Beyer E.C. (2003). Plasma membrane channels formed by connexins: Their regulation and functions. Physiol. Rev..

[13] Sohl G., Willecke K. (2003). An update on connexin genes and their nomenclature in mouse and man. Cell Commun. Adhes..

[14] Beyer E.C., Paul D.L., Goodenough D.A. (1987). Connexin43: A protein from rat heart homologous to a gap junction protein from liver. J. Cell Biol..

[15] Retamal M.A., Froger N., Palacios-Prado N., Ezan P., Sáez P.J., Sáez J.C., Giaume C. (2007). Cx43 hemichannels and gap junction channels in astrocytes are regulated oppositely by proinflammatory cytokines released from activated microglia. J. Neurosci..

[16] Orellana J.A., Hernández D.E., Ezan P., Velarde V., Bennett M.V., Giaume C., Sáez J.C. (2010). Hypoxia in high glucose followed by reoxygenation in normal glucose reduces the viability of cortical astrocytes through increased permeability of connexin 43 hemichannels. Glia.

[17] Bruzzone R., Hormuzdi S.G., Barbe M.T., Herb A., Monyer H. (2003). Pannexins, a family of gap junction proteins expressed in brain. Proc. Natl. Acad. Sci. USA.

[18] Sosinsky G.E., Boassa D., Dermietzel R., Duffy H.S., Laird D.W., MacVicar B., Naus C.C., Penuela S., Scemes E., Spray D.C. (2011). Pannexin channels are not gap junction hemichannels. Channels (Austin).

[19] Volonte C., Apolloni S., Skaper S.D., Burnstock G. (2012). P2X7 receptors: Channels, pores and more. CNS Neurol. Disord. Drug Targets.

[20] Pelegrin P., Surprenant A. (2006). Pannexin-1 mediates large pore formation and interleukin-1beta release by the ATP-gated P2X7 receptor. EMBO J..

[21] Hanner F., Lam L., Nguyen M.T., Yu A., Peti-Peterdi J. (2012). Intrarenal localization of the plasma membrane ATP channel pannexin1. Am. J. Physiol. Ren. Physiol..

[22] Kurtz A. (2012). Renal connexins and blood pressure. Biochim. Biophys. Acta.

[23] Menzies R.I., Howarth A.R., Unwin R.J., Tam F.W., Mullins J.J., Bailey M.A. (2015). Inhibition of the purinergic P2X7 receptor improves renal perfusion in angiotensin-II-infused rats. Kidney Int..

[24] Haefliger J.A., Krattinger N., Martin D., Pedrazzini T., Capponi A., Doring B., Plum A., Charollais A., Willecke K., Meda P. (2006). Connexin43-dependent mechanism modulates renin secretion and hypertension. J. Clin. Investig..

[25] Hillis G.S., Duthie L.A., Mlynski R., McKay N.G., Mistry S., MacLeod A.M., Simpson J.G., Haites N.E. (1997). The expression of connexin 43 in human kidney and cultured renal cells. Nephron.

[26] Vonend O., Turner C.M., Chan C.M., Loesch A., Dell’Anna G.C., Srai K.S., Burnstock G., Unwin R.J. (2004). Glomerular expression of the ATP-sensitive P2X receptor in diabetic and hypertensive rat models. Kidney Int..

[27] Vergara L., Bao X., Cooper M., Bello-Reuss E., Reuss L. (2003). Gap-junctional hemichannels are activated by ATP depletion in human renal proximal tubule cells. J. Membr. Biol..

[28] Schalper K.A., Palacios-Prado N., Orellana J.A., Sáez J.C. (2008). Currently used methods for identification and characterization of hemichannels. Cell Commun. Adhes..

[29] Haas M.J., Onstead-Haas L., Lee T., Torfah M., Mooradian A.D. (2016). Angiotensin II receptor one (AT1) mediates dextrose induced endoplasmic reticulum stress and superoxide production in human coronary artery endothelial cells. Int. J. Cardiol..

[30] Orellana J.A., Sáez P.J., Shoji K.F., Schalper K.A., Palacios-Prado N., Velarde V., Giaume C., Bennett M.V., Sáez J.C. (2009). Modulation of brain hemichannels and gap junction channels by pro-inflammatory agents and their possible role in neurodegeneration. Antioxid. Redox Signal..

[31] Shoji K.F., Sáez P.J., Harcha P.A., Aguila H.L., Sáez J.C. (2014). Pannexin1 channels act downstream of P2X 7 receptors in ATP-induced murine T-cell death. Channels (Austin).

[32] Hanner F., Sorensen C.M., Holstein-Rathlou N.H., Peti-Peterdi J. (2010). Connexins and the kidney. Am. J. Physiol. Regul. Integr. Comp. Physiol..

[33] Warner A., Clements D.K., Parikh S., Evans W.H., DeHaan R.L. (1995). Specific motifs in the external loops of connexin proteins can determine gap junction formation between chick heart myocytes. J. Physiol..

[34] Stehberg J., Moraga-Amaro R., Salazar C., Becerra A., Echeverria C., Orellana J.A., Bultynck G., Ponsaerts R., Leybaert L., Simon F. (2012). Release of gliotransmitters through astroglial connexin 43 hemichannels is necessary for fear memory consolidation in the basolateral amygdala. FASEB J..

[35] Dahl G., Qiu F., Wang J. (2013). The bizarre pharmacology of the ATP release channel pannexin1. Neuropharmacology.

[36] Bhaskaracharya A., Dao-Ung P., Jalilian I., Spildrejorde M., Skarratt K.K., Fuller S.J., Sluyter R., Stokes L. (2014). Probenecid blocks human P2X7 receptor-induced dye uptake via a pannexin-1 independent mechanism. PLoS ONE.

[37] Orellana J.A., Diaz E., Schalper K.A., Vargas A.A., Bennett M.V., Sáez J.C. (2011). Cation permeation through connexin 43 hemichannels is cooperative, competitive and saturable with parameters depending on the permeant species. Biochem. Biophys. Res. Commun..

[38] Nakazawa K., Liu M., Inoue K., Ohno Y. (1997). Potent inhibition by trivalent cations of ATP-gated channels. Eur. J. Pharmacol..

[39] Hernández-Salinas R., Vielma A.Z., Arismendi M.N., Boric M.P., Sáez J.C., Velarde V. (2013). Boldine prevents renal alterations in diabetic rats. J. Diabetes Res..

[40] Feng Z., Wei C., Chen X., Wang J., Cheng H., Zhang X., Hong Q., Shi S., Fu B., Wei R. (2006). Essential role of Ca^2+^ release channels in angiotensin II-induced Ca^2+^ oscillations and mesangial cell contraction. Kidney Int..

[41] Qiu G., Ji Z. (2014). AngII-induced glomerular mesangial cell proliferation inhibited by losartan via changes in intracellular calcium ion concentration. Clin. Exp. Med..

[42] Zhao W., Chen S.S., Chen Y., Ahokas R.A., Sun Y. (2008). Kidney fibrosis in hypertensive rats: Role of oxidative stress. Am. J. Nephrol..

[43] Schalper K.A., Sánchez H.A., Lee S.C., Altenberg G.A., Nathanson M.H., Sáez J.C. (2010). Connexin 43 hemichannels mediate the Ca^2+^ influx induced by extracellular alkalinization. Am. J. Physiol. Cell Physiol..

[44] Fiori M.C., Figueroa V., Zoghbi M.E., Sáez J.C., Reuss L., Altenberg G.A. (2012). Permeation of calcium through purified connexin 26 hemichannels. J. Biol. Chem..

[45] Van Kats J.P., de Lannoy L.M., Jan Danser A.H., van Meegen J.R., Verdouw P.D., Schalekamp M.A. (1997). Angiotensin II type 1 (AT1) receptor-mediated accumulation of angiotensin II in tissues and its intracellular half-life in vivo. Hypertension.

[46] Reuveny M., Heller H., Bengal E. (2004). RhoA controls myoblast survival by inducing the phosphatidylinositol 3-kinase-Akt signaling pathway. FEBS Lett..

[47] Puebla C., Cisterna B.A., Salas D.P., Delgado-López F., Lampe P.D., Sáez J.C. (2016). Linoleic acid permeabilizes gastric epithelial cells by increasing connexin 43 levels in the cell membrane via a GPR40- and Akt-dependent mechanism. Biochim. Biophys. Acta.

[48] De Vuyst E., Wang N., Decrock E., De Bock M., Vinken M., Van Moorhem M., Lai C., Culot M., Rogiers V., Cecchelli R. (2009). Ca^2+^ regulation of connexin 43 hemichannels in C6 glioma and glial cells. Cell Calcium.

[49] Zhang M., Piskuric N.A., Vollmer C., Nurse C.A. (2012). P2Y2 receptor activation opens pannexin-1 channels in rat carotid body type II cells: potential role in amplifying the neurotransmitter ATP. J. Physiol..

[50] Cisterna B.A., Vargas A.A., Puebla C., Sáez J.C. (2016). Connexin hemichannels explain the ionic imbalance and lead to atrophy in denervated skeletal muscles. Biochim. Biophys. Acta.

[51] Liu L., Hu X., Cai G.Y., Lv Y., Zhuo L., Gao J.J., Cui S.Y., Feng Z., Fu B., Chen X.M. (2012). High glucose-induced hypertrophy of mesangial cells is reversed by connexin43 overexpression via PTEN/Akt/mTOR signaling. Nephrol. Dial. Transplant..

[52] Hayashi K., Wakino S., Kanda T., Homma K., Sugano N., Saruta T. (2006). Molecular mechanisms and therapeutic strategies of chronic renal injury: role of rho-kinase in the development of renal injury. J. Pharmacol. Sci..

[53] Anderson S.C., Stone C., Tkach L., SundarRaj N. (2002). Rho and Rho-kinase (ROCK) signaling in adherens and gap junction assembly in corneal epithelium. Investig. Ophthalmol. Vis. Sci..

[54] Langevin H.M., Fujita T., Bouffard N.A., Takano T., Koptiuch C., Badger G.J., Nedergaard M. (2013). Fibroblast cytoskeletal remodeling induced by tissue stretch involves ATP signaling. J. Cell. Physiol..

[55] Orellana J.A., Sáez P.J., Cortés-Campos C., Elizondo R.J., Shoji K.F., Contreras-Duarte S., Figueroa V., Velarde V., Jiang J.X., Nualart F. (2012). Glucose increases intracellular free Ca^2+^ in tanycytes via ATP released through connexin 43 hemichannels. Glia.

[56] Saffitz J.E., Laing J.G., Yamada K.A. (2000). Connexin expression and turnover: Implications for cardiac excitability. Circ. Res..

[57] Retamal M.A., Cortés C.J., Reuss L., Bennett M.V., Sáez J.C. (2006). S-nitrosylation and permeation through connexin 43 hemichannels in astrocytes: Induction by oxidant stress and reversal by reducing agents. Proc. Natl. Acad. Sci. USA.

[58] Figueroa X.F., Lillo M.A., Gaete P.S., Riquelme M.A., Sáez J.C. (2013). Diffusion of nitric oxide across cell membranes of the vascular wall requires specific connexin-based channels. Neuropharmacology.

[59] Miyata K., Satou R., Shao W., Prieto M.C., Urushihara M., Kobori H., Navar L.G. (2014). ROCK/NF-kappaB axis-dependent augmentation of angiotensinogen by angiotensin II in primary-cultured preglomerular vascular smooth muscle cells. Am. J. Physiol. Ren. Physiol..

[60] Ishikawa Y., Nishikimi T., Akimoto K., Ishimura K., Ono H., Matsuoka H. (2006). Long-term administration of rho-kinase inhibitor ameliorates renal damage in malignant hypertensive rats. Hypertension.

[61] Cea L.A., Cisterna B.A., Puebla C., Frank M., Figueroa X.F., Cardozo C., Willecke K., Latorre R., Sáez J.C. (2013). De novo expression of connexin hemichannels in denervated fast skeletal muscles leads to atrophy. Proc. Natl. Acad. Sci. USA.

[62] Willebrords J., Cogliati B., Pereira I.V.A., da Silva T.C., Crespo Yanguas S., Maes M., Govoni V.M., Lima A., Felisbino D.A., Decrock E. (2017). Inhibition of connexin hemichannels alleviates non-alcoholic steatohepatitis in mice. Sci. Rep..

[63] Mugisho O.O., Green C.R., Kho D.T., Zhang J., Graham E.S., Acosta M.L., Rupenthal I.D. (2018). The inflammasome pathway is amplified and perpetuated in an autocrine manner through connexin43 hemichannel mediated ATP release. Biochim. Biophys. Acta.

[64] Ma R., Chaudhari S., Li W. (2016). Canonical Transient Receptor Potential 6 Channel: A New Target of Reactive Oxygen Species in Renal Physiology and Pathology. Antioxid. Redox Signal..

[65] Riquelme M.A., Cea L.A., Vega J.L., Boric M.P., Monyer H., Bennett M.V., Frank M., Willecke K., Sáez J.C. (2013). The ATP required for potentiation of skeletal muscle contraction is released via pannexin hemichannels. Neuropharmacology.

[66] Contreras J.E., Sánchez H.A., Eugenín E.A., Speidel D., Theis M., Willecke K., Bukauskas F.F., Bennett M.V., Sáez J.C. (2002). Metabolic inhibition induces opening of unapposed connexin 43 gap junction hemichannels and reduces gap junctional communication in cortical astrocytes in culture. Proc. Natl. Acad. Sci. USA.

[67] Lowry O.H., Rosebrough N.J., Farr A.L., Randall R.J. (1951). Protein measurement with the Folin phenol reagent. J. Biol. Chem..

[68] Ramanathan L., Das N.P., Li Q.T. (1994). Studies on lipid oxidation in fish phospholipid liposomes. Biol. Trace Elem. Res..

